# Accounting for Individual Differences in Decision-Making Competence: Personality and Gender Differences

**DOI:** 10.3389/fpsyg.2018.02258

**Published:** 2018-11-23

**Authors:** Joshua Weller, Andrea Ceschi, Lauren Hirsch, Riccardo Sartori, Arianna Costantini

**Affiliations:** ^1^Department of Developmental Psychology, Tilburg School of Social and Behavioral Sciences, Tilburg University, Tilburg, Netherlands; ^2^Department of Human Sciences, University of Verona, Verona, Italy; ^3^Oregon State University, Corvallis, OR, United States

**Keywords:** HEXACO, HEXACO Honesty/Humility, conscientiousness, decision-making competence, decision-making, individual differences, gender differences

## Abstract

Emerging research has highlighted the utility of measuring individual differences in decision-making competence (DMC), showing that consistently following normatively rational principles is associated with positive psychosocial and health behaviors. From another level of analysis, functional theories of personality suggest that broad trait dimensions represent variation in underlying self-regulatory systems, providing a mechanistic account for robust associations between traits and similar life outcomes. Yet, the degree to which broad dispositional personality dimensions predict global tendencies to respond rationally is less understood. In a large online community sample (*N* = 804), we tested the associations between HEXACO personality dimensions, a 6-factor structural trait model, and a subset of DMC indicators (Applying Decision Rules, Resistance to Framing, Recognizing Social Norms, and Consistency in Risk Perception). Additionally, we examined gender differences across the DMC, first considering the potential for measurement non-invariance across groups for the DMC. We observed partial measurement invariance between men and women; only the Applying Decision Rules scale showed evidence of differential functioning across groups. Controlling for these differences, analyses revealed that higher Conscientiousness, Honesty/Humility, and Openness were associated with higher DMC scores. In contrast, Emotionality and Extraversion demonstrated gender-specific associations. Specifically, low Extraversion was associated with higher DMC scores for men, whereas higher Emotionality was associated with higher DMC scores for women. Our results suggest that traits related to self-regulatory functions of cognitive and behavioral control, and cognitive flexibility are associated with an increased tendency to engage in rational thought.

## Introduction

Each day, the decisions that we make have the potential to directly impact our health, happiness, and well-being. It follows that the ability to make sound, “quality” choices is likely to promote positive outcomes and minimize the possibility of negative ones. However, what makes a “quality” decision, and why do people often fail to make them? Since Kahneman and Tversky’s (1972) seminal investigations of heuristics and biases, researchers have acknowledged that human decision processes often deviate from predictions made from normative models of rationality (e.g., Expected Utility Theory, von Neumann and Morgenstern, 1947). Instead of employing a rational analysis, individuals frequently base their choices on a subset of information available, limited information searches, and preferences that may switch as a result of changes in how irrelevant attributes of the available choice options are presented. This “bounded rationality” is believed to be a cognitive adaptation developed to best make efficient and accurate choices in light of cognitive processing limitations, such as limited working memory (Simon, 1991). Though this efficient, non-compensatory approach often leads to a satisfactory choice, the indiscriminate application of mental shortcuts can lead to judgmental errors that may bear significant costs to well-being, finances, and society at-large (Hastie and Dawes, 2010).

Emerging research suggests that stable individual differences in the tendency to respond rationally are associated with positive health and financial outcomes (e.g., Parker et al., 2018). These results complement similar evidence of predictive validity observed in the personality literature. Specifically, broad dimensions of personality, such as those recovered in structural models like the “Big 5” and HEXACO (e.g., Goldberg, 1993; Ashton and Lee, 2007) predict life outcomes that are believed to be, in part, related to advantageous (and disadvantageous) decision processes (e.g., educational attainment, workplace performance and behaviors, substance use, antisociality; Roberts et al., 2007). In fact, new perspectives in personality theory view traits as phenotypic expressions of the efficiency of broader self-regulatory systems that subserve the decisions that we make (DeYoung, 2015; Wood and Denissen, 2015). However, associations between rational responding and broad-level personality dimensions largely have not been well-explicated. Prior studies have examined the associations between decision behavior and personality, but often have done so with a single decision paradigm, a narrow range of traits, and/or with small sample sizes that raise the possibility for non-replication of effects (e.g., Levin et al., 2002; Lauriola et al., 2005; Parker and Fischhoff, 2005).

The current study aimed to address this gap in the literature. Specifically, we tested the degree to which personality traits were associated with individual differences in decision-making competence (DMC), a construct that aims to quantify individual differences in rational responding across six decision domains (e.g., Parker and Fischhoff, 2005; Bruine de Bruin et al., 2007; Parker et al., 2018). Additionally, we considered these associations in light of mixed evidence suggesting potential gender differences with respect to DMC performance. For instance, men self-report engaging in more risk-taking behaviors, but typically show better performance on laboratory-based decision-making paradigms (e.g., Byrnes et al., 1999b; Weller et al., 2010). Similarly, whereas men perform better in laboratory tasks involving probabilistic reasoning, these effects appear less strong, or even absent, when a decision does not heavily recruit this process (Stanovich et al., 2017). Establishing these associations has the potential to not only illuminate *who* makes suboptimal decisions, but also *why* certain individuals may engage in poorer real-life decision outcomes.

### Assessing Individual Differences in Rational Responding

Researchers have increasingly acknowledged considerable individual differences in the tendency to respond in a manner consistent with predictions made from normative models of rationality, such as Expected Utility Theory (Lauriola and Levin, 2001; LeBoeuf and Shafir, 2003; Levin et al., 2002; von Neumann and Morgenstern, 1947; West et al., 2008). These tendencies appear to be interrelated across tasks, suggesting that reasoning errors are not solely context-dependent, but instead, driven by a set of common cognitive mechanisms (Stanovich et al., 2017). This insight has led to the development of assessment batteries comprised of decision-making tasks that aim to quantify an overall aggregate of individual differences in rational responding (Parker and Fischhoff, 2005; Bruine de Bruin et al., 2007; Halpern, 2010; Stanovich et al., 2017).

In particular, DMC assessment batteries include several indicators of decision quality, using tasks that compare an individual’s response to that predicted by a normative standard. Such standards can be met either via (a) *coherence*, representing response consistency across multiple presentations of objectively equivalent, but differently presented, decision problems (i.e., risky-choice framing tasks; Tversky and Kahneman, 1981), or (b) *correspondence* (i.e., accuracy of an individual’s response compared to an objectively correct answer, such as the ability to follow a series of rules to select a correct option out of a multi-attribute matrix; Payne et al., 1993). As an objective performance test, DMC can be distinguished from self-reported measures of perceived decision-competence, which tap self-efficacy beliefs (Byrnes et al., 1999a; see Weller et al., 2013 for a similar contrast in the domain of numeracy). Also, though there has been a link between cognitive styles (e.g., polarized thinking, need for cognition), and both DMC and personality (Parker and Fischhoff, 2005; Bruine de Bruin et al., 2007; Delaney et al., 2015; Volkova and Rusalov, 2016), we consider the former a descriptive construct that highlights dispositional tendencies to process information (e.g., “I am the type of person who likes to have as much information as possible before making a decision”). However, although certain cognitive styles may indeed be related to a greater tendency to respond rationally, cognitive style scales do not directly tap into these tendencies. Additionally, it is possible that an individual may process decision information deliberately, but apply “contaminated mindware,” or biased and faulty premises about the world, which subsequently may lead to non-normative responding (Stanovich et al., 2017). Finally, DMC can be separated from measures that aim to reflect everyday decision quality in terms of behavioral outcomes, such as the Decision Outcomes Inventory (DOI; Bruine de Bruin et al., 2007; Dewberry et al., 2013; Geisler and Allwood, 2015). Whereas a measure like the DOI reflects outcomes that may or may not be the result of a rational decision process, the DMC construct focuses on the adequacy of the choice itself (i.e., rational responding), independent of outcomes. This difference is important because even though a quality decision (from a rational decision-making perspective) was made, the desired outcome may not be realized, and vice versa.

Prior studies have demonstrated convergent validity between DMC and neurocognitive mechanisms believed to promote advantageous decision-making, such as executive function, working memory, and inhibitory control, and other decision skills (Parker and Fischhoff, 2005; Bruine de Bruin et al., 2007; Del Missier et al., 2012; Parker and Weller, 2015; Weller et al., 2015b; Parker et al., 2018). Additionally, higher DMC scores appear to correlate with the likelihood to desist from engaging in problematic behaviors. For instance, Parker and Fischhoff (2005) found that lower DMC scores predicted higher incidence of delinquency, substance abuse and health-risking sexual behaviors (c.f., Weller et al., 2015a). Further, Bruine de Bruin et al. (2007) observed that low DMC scores were associated with an increased tendency to report negative outcomes across a variety of domains. Similarly, Weller et al. (2015a) reported that lower DMC scores, measured at age 10–11, predicted increased conduct, emotional, and peer problems, as well as lower self-reported pro-social tendencies 2 years after the initial DMC assessment.

### Personality and Decision-Making Competence

Similar to the associations between DMC and potentially problematic life outcomes, research also has demonstrated robust associations between broad-spectrum trait dimensions and life outcomes (Arthur and Graziano, 1996; Bogg and Roberts, 2004; Roberts et al., 2007). Functional accounts of personality posit that these associations exist because traits represent a collection of affective, behavioral, and cognitive tendencies that (a) reflect strategic means by which an individual achieves a desired goal state, and, more broadly, (b) variation in the efficiency and effectiveness of a broader self-regulatory system (Denissen et al., 2013; DeYoung, 2015; Fleeson and Jayawickreme, 2015). For instance, DeYoung (2015) cybernetic Big 5 personality model, integrates broad personality traits within the context of a cybernetic systems model (i.e., the study of goal-directed, self-regulating systems; Wiener, 1961; Austin and Vancouver, 1996; Carver and Scheier, 1998; DeYoung, 2015). In this model, broad trait dimensions represent particular functions that help one move through a cybernetic chain, from (a) goal activation to (b) action selection, (c) action, (d) outcome interpretation, and finally, (e) comparison of the outcome to initial goal state. Moreover, broad traits may impact this process at multiple stages. For instance, a conscientious person may be more likely to activate an achievement-oriented goal of passing an exam instead of an affiliation-goal of spending time with friends (i.e., goal activation), choose to stay at home to study instead of going out to a party (i.e., action selection), and decide to study instead of surfing the internet (i.e., action).

Although DeYoung’s model highlighted the functions of Big 5 traits, its insights also are applicable to other phenotypic structural models of personality, such as the HEXACO (Ashton and Lee, 2007), due to the large overlap between these models. Similar to the “Big Five” model of personality, the HEXACO also includes the trait dimensions of Extraversion, Conscientiousness, Agreeableness, Openness, and Emotionality (akin to Neuroticism); for these traits, the content coverage within each dimension is similar enough at the broadest level to be roughly equivalent when comparing research across models. However, the most important advantage for using the HEXACO structure is that it also recovers a 6th factor, Honesty/Humility, which represents a tendency to adhere to social norms and traditions, approach interpersonal interactions with sincerity and fairness, and not expect special treatment from others (Ashton and Lee, 2007). Notably, some studies have demonstrated superior predictive validity for the HEXACO Honesty/Humility dimension over the Big 5 across numerous domains (Ashton and Lee, 2008). We focused our hypotheses on three dimensions that may be the most strongly associated with rational responding: Conscientiousness, Openness, and Honesty/Humility.

#### Conscientiousness

Conscientiousness reflects variation in the mechanisms that drive tendencies to be organized, set goals, and work toward them in an ordered way. Conscientious individuals are more likely to pursue academic and achievement-oriented goals (Roberts and Robins, 2000), and also demonstrate stronger performance in academic and job settings (Schmidt and Hunter, 1998; Barrick et al., 2001; de Vries et al., 2011). Lower Conscientiousness has been associated with a wide variety of risk-taking behaviors, which may adversely affect long-term health and psychosocial outcomes (Gullone and Moore, 2000; Terracciano and Costa, 2004; Weller and Tikir, 2011). When goals have been set, greater Conscientiousness may facilitate staying on task and not being distracted by non-primary incentives or information irrelevant to the task. Neuroimaging studies have demonstrated associations between Conscientiousness and neural regions associated with attention, goal-directed planning, and cognitive control (DeYoung et al., 2010; Adelstein et al., 2011). Directly related to individual differences in DMC, Weller et al. (2012) found that preadolescent children with higher levels of Effortful Control scores, a temperament trait related to conscientiousness in adulthood (Rothbart and Ahadi, 1994), performed better on a subset of DMC scales.

#### Openness

Individuals reporting higher Openness to Experience tend to be inquisitive, use their imagination, and take interest in others’ points of view (Ashton and Lee, 2007). According to DeYoung (2014, 2015), Openness corresponds to individual differences in cognitive exploration and engagement with information. These functions may help to detect discrepancies between the current state and the desired state identify goal-relevant stimuli in the environment, and predict what strategies might be most effective for goal pursuit (DeYoung, 2015; p. 44). Moreover, individuals who report higher Openness to Experience may experience more intrinsic rewards for engaging in cognitive activity, which may also represent a motivational factor for remaining engaged when faced with complex decisions (Denissen and Penke, 2008). Additionally, functions ascribed to Openness, such as taking into consideration other’s points of view and actively seeking out new information, are vital skills needed to make advantageous decisions, especially in social situations (Hastie and Dawes, 2010). Conversely, lower Openness may lead an individual to make biased judgments based on a limited subset of information, or on current emotions (Stanovich and West, 2008; Haran et al., 2013). Finally, Baron (1993) suggests that “actively open-minded thinkers,” a construct that shares variance with both Openness and Conscientiousness, are more likely to seek out, attend to, and evaluate a more complete set of information when making choices. West et al. (2008) found that greater actively open-minded thinking was associated with greater resistance to a variety of heuristics and biases (Stanovich and West, 2007).

#### Honesty/Humility

Within a functional framework, greater Honesty/Humility may represent self-regulatory tendencies that help one avoid acting solely upon egoistic, self-interested impulses, as well as the willingness to exploit others for personal gain. This dimension may most likely be associated with normative decision making, not only when the task involves understanding others (i.e., accurately detecting social norms), but also when short-term gains may be at stake. Lower Honesty/Humility has been associated with greater self-reported ethical and health-related risk-taking (de Vries et al., 2009; Ashton et al., 2010; Weller and Tikir, 2011), higher rates of counterproductive workplace behaviors (Lee et al., 2005) and lower academic outcomes (Taylor et al., 2015). Similarly, lower Honesty/Humility has been associated with impulsivity and the “dark triad” traits (Psychopathy, Machiavellianism, and Narcissism), traits that are associated with antisocial tendencies and lower cognitive control (Lee and Ashton, 2005; de Vries et al., 2009; Witt et al., 2009). With respect to decision-making tendencies, Taylor et al. (2015) found that higher Honesty/Humility was associated with fewer errors on a conditional reasoning task, suggesting that these individuals responded more rationally on this task. Geisler and Allwood (2018) also reported similar, albeit indirect, evidence for the association between Honesty/Humility and DMC. Specifically, they found that individuals reporting higher Machiavellianism demonstrated lower DMC performance.

### Gender Differences

In order to gain a clearer conceptualization of the associations between traits and DMC, we first considered potential gender differences. Reported gender differences in decision-making may represent biological differences in neuropsychological processes (van de Bos et al., 2013), socialization processes (Eagly, 1995), or a combination of both. Regardless of the underlying etiology, such differences may result in behavioral and motivational differences that potentially would be impactful for the goals that are set and the manner by which they are enacted.

With respect to personality, evidence suggests small gender differences overall for Extraversion, Conscientiousness, Openness, and Agreeableness, but medium to large differences for Neuroticism and Honesty/Humility (i.e., women report higher scores than men; Lee and Ashton, 2004; Schmitt et al., 2008; Wakabayashi, 2014; Romero et al., 2015). In contrast, research has yielded mixed results regarding gender differences for skills believed to be related to DMC. For instance, women tend to demonstrate lower sensitivity to expected value, indicative of less rational responding^1^ in uncertainty-based laboratory decision-making tasks (Weller et al., 2010). Similarly, men tend to score higher than women on numeracy and cognitive reflection, tendencies believed to support mechanisms of advantageous decision-making (Frederick, 2005; Weller et al., 2013; Stanovich et al., 2017). Moreover, Toplak et al. (2017) found men performed better than women on a composite “heuristic and bias” index, which included items related to cognitive reflection, in addition to statistical knowledge and syllogistic reasoning. However, gender differences do not appear to be consistent across different competencies, leaving this an open question. For instance, Stanovich et al. (2017) reported no gender differences for framing, risk knowledge, and overconfidence measures.

Surprisingly, to our knowledge, gender differences for DMC performance infrequently have been reported. In fact, only two studies have reported gender differences, with varying results. In a study that aimed to validate the Adult-DMC scale in a Slovak sample of high school and university students, Bavolar (2013) reported that men outperformed women on the Applying Decision Rules and Sunk Costs scales only. In contrast, in another validation study with a Chinese sample of undergraduate students, Liang and Zou (2018) reported no significant differences for Applying Decision Rules, but did find similar gender differences for sunk cost effects. Notably, neither study found significant gender differences for Resistance to Framing Effects or Under/Overconfidence (c.f., Stanovich et al., 2017).

These mixed results across different language versions of the task raise the issue of potential measurement non-invariance across groups (i.e., two individuals with the same ability level on an underlying latent trait should have the same test or item score). Specifically, if non-invariance is present, mean group differences on a latent trait, or lack thereof, may result from underlying differential functioning of the indicators across groups (i.e., test bias) rather than actual differences in latent trait scores. In turn, subsequent research that would report validity coefficients based on a common regression line may inaccurately estimate the magnitude of the effect for one, or both groups, and therefore, raises questions related to interpretability of a test score.

Though the current study was not designed to evaluate cross-cultural differences in DMC, it can evaluate measurement invariance across gender within a culture. Naturally, inquiries regarding gender differences for DMC performance should first be subject to measurement invariance testing across groups within a specific culture. Invariance testing allows researchers to identify potential sources of bias, and control/correct for them (Vandenberg and Lance, 2000). However, the degree to which DMC demonstrates measurement invariance remains an empirical question. Invariance testing for gender differences on intelligence tests has typically demonstrated at least partial measurement invariance for both adults and children (Dolan et al., 2006; Chen et al., 2015). Yet, individual differences in rational responding, and more specifically DMC performance, are considered to be separable from general mental ability (Stanovich et al., 2017); thus, it is imprudent to extend invariance results from one construct to another.

### Hypotheses

In this study, we made three specific predictions. First, we predicted that higher levels of Conscientiousness will be related to higher DMC scores, and that this trait would be most strongly associated with DMC. Second, we predicted that higher self-reported Openness would be positively correlated with DMC scores. Third, we expected that higher self-reported Honesty/Humility would be associated with higher DMC scores. With respect to gender, prior research does not clearly suggest a consistent pattern of differences across the skills assessed by the DMC components, making it imprudent to propose an overall directional hypothesis, though the content of our tasks compared to the tasks described in Stanovich et al. (2017) would suggest that men would outperform women on an overall DMC performance score if probabilistic knowledge is heavily recruited on these scales. Similarly, to our knowledge, invariance testing has never been conducted on an index of rational responding before, leaving us without prior results by which we could base a hypothesis. Thus, we considered these research questions to be more exploratory in nature.

## Materials and Methods

### Participants

A third-party research firm sent 7044 participant invitation emails to an opt-in panel of Italian community residents. 921 subjects completed the entire survey. We excluded 50 participants who took less than 10 min to complete the entire survey (Median = 31.78 min), and an additional 67 subjects because they demonstrated evidence of careless responding (e.g., stylistic or identical response patterns or overtly careless responses across equivalently worded, but reversed items throughout the assessment), resulting in *N* = 804.

The mean age for the final sample was 34.96 years (*SD* = 8.24; 58% women). Males were slightly older (*M* = 35.87 years, *SD* = 8.10) than females (*M* = 34.37, *SD* = 8.30), *t(802)* = 2.47, *p* = 0.014. Of those who completed the study, 7.2% of participants did not possess a high school diploma, 52.2% had a high school diploma or equivalent, 21.4% received a bachelor’s degree, and 19.1% received an advanced college degree. A chi-square analysis testing for gender differences in education level (High School education or less = 0; More than high school education = 1) was not significant (χ^2^ = 0.27, *p* = 0.61). This study was approved by the supporting university’s Ethical Review Committee.

### Measures

#### Adult Decision-Making Competence (A-DMC)

Participants completed four scales (Resistance to Framing, Consistency in Risk Perception, Applying Decision Rules, and Recognizing Social Norms) from the Italian-language A-DMC assessment (Del Missier et al., 2012). The scale intercorrelations are reported in Supplementary Information S1.

#### Resistance to Framing

Resistance to Framing was operationalized as the consistency of responding across fourteen equivalent, but opposite-framed, item pairs. Respondents either (a) made a choice between a risky versus riskless option (i.e., risky choice-framing items), or (b) rated the favorability of an event/product based on a framed-attribute (i.e., attribute framing). Choices were made on a six-point Likert scale (e.g., 1 = definitely prefer option A; 6 = definitely prefer option B). Framing resistance was determined by first calculating difference scores for the equivalent pairs. Then, a mean absolute composite score was calculated. Scores were then reflected such that greater positive values related to more Resistance to Framing, α = 0.67. Other components of this larger study were administered between different versions of the framing problems to maximally space the differently framed presentations.

#### Applying Decision Rules

This 10-item measure assessed participants’ ability to follow a set of rules in order to make an accurate selection from five options in a multi-attribute matrix. Participants were asked to select a DVD system that matched a hypothetical buyer’s search criteria (e.g., “Paolo wants to buy the DVD with the most attribute ratings that were above average.”). For each scenario, participants chose from a different set of five equally priced DVD players with varying ratings of picture quality, sound quality, programming options, and brand reliability (1 = *very low*; 5 = *very high*). Performance was measured by the number of total correct scores, α = 0.65.

#### Consistency in Risk Perception

Across 10 item pairs, we assessed the tendency for participants to follow a basic rule of probability judgment (i.e., an event has a greater probability of occurring over a longer time frame than a shorter one). Specifically, respondents were first asked to rate the possibility of an event occurring to them within the next month (e.g., What is the probability that your driving will be accident-free?). Later in the assessment, participants evaluated the probability that the same events would occur to them over the next 2 years^2^. Performance was indicated by the number of probability-consistent responses made, α = 0.57.

#### Recognizing Social Norms

For this scale, participants first evaluated the opportunity to endorse whether or not it is “sometimes OK” to engage in several behaviors that may be deemed undesirable (e.g., to steal under certain circumstances). Later in the assessment, respondents were asked to rate “out of 100 people your age,” how many would endorse each of the same behaviors (i.e., peer endorsement). Performance was measured by each individual’s correlation between the actual endorsement rate of the behavior in the sample (out of 100%) and their estimated percentage of perceived peer endorsements across the 16 behaviors, α = 0.76 for individual endorsement rates.

### Personality Dimensions

#### HEXACO-PI-R

Participants completed the Italian-language version of the 60-item HEXACO-PI-R^3^ (Ashton et al., 2006; Ashton and Lee, 2009). Each item was rated on a five-point Likert scale (5 = strongly agree; 1 = strongly disagree). The scale measures six broad personality dimensions: Honesty/Humility, Emotionality, Extraversion, Agreeableness, Conscientiousness, and Openness (range α = 0.87–0.90). Intercorrelations between the HEXACO dimensions ranged between *r* = -0.15 (between Extraversion and Emotionality) to *r* = 0.38 (between Extraversion and Openness). Intercorrelations of the HEXACO dimensions are presented in Supplementary Information S2.

## Results

### Mean Level Gender Differences for HEXACO and DMC

As shown in Table 1, we observed the strongest gender differences for HEXACO dimensions of Emotionality and Honesty/Humility, in which women reported higher levels of each (*d* = 0.62; *d* = 0.34). In contrast, higher Agreeableness was reported for men (*d* = 0.23). The other dimensions showed small effects (|*d*| < 0.20), which were all significant at *p* < 0.05 with the exception of Extraversion. Concerning DMC component measures, males performed better than females on the Applying Decision Rules and Resistance to Framing scales, *t*(802) = 2.38, *p* = 0.018 and 2.26, *p* = 0.027, respectively.

**Table 1:** Descriptive statistics of HEXACO personality dimensions and DMC components.

	Women		Men		Gender differences Cohen’s |*d*|
	
	*M*	*SD*	*M*	*SD*	
**HEXACO Scale**			
Honesty-Humility	3.50	0.60	3.30	0.59	0.34
Emotionality	3.33	0.56	3.00	0.49	0.63
Extraversion	3.30	0.60	3.23	0.55	0.12
Agreeableness	2.97	0.54	3.09	0.52	0.23
Conscientiousness	3.67	0.57	3.57	0.58	0.18
Openness to experience	3.50	0.62	3.40	0.60	0.17
**DMC component (*z*-scored)**			
Recognizing social norms	0.05	0.97	-0.08	1.04	0.13
Resistance to framing	-0.06	0.99	0.10	1.01	0.16
Applying decision rules	-0.07	0.94	0.10	1.04	0.17
Consistency in risk perception	0.04	0.97	-0.06	1.04	0.10

### Correlations Between HEXACO Dimensions and DMC Components

Table 2 reports the correlations between DMC component performance and the HEXACO dimensions. We did not find a robust pattern of correlations between age and DMC scores; considering that this study was not designed to study age differences, we do not discuss age further. Honesty/Humility, Conscientiousness, and Openness to Experience were positively associated with performance on DMC components. We note two exceptions to this pattern. First, unlike the other DMC components in the overall sample, Resistance to Framing was largely uncorrelated with the HEXACO dimensions, with the exception that greater Resistance to Framing was associated with lower Extraversion. Second, we observed notable divergent patterns when examining gender-specific correlations. For women, Recognizing Social Norms and Consistency in Risk Perception were positively associated with Emotionality, but this pattern was not present for men. In contrast, higher Honesty/Humility was positively associated with greater Resistance to Framing for men, but not for women.

**Table 2:** Correlations between HEXACO personality dimensions and DMC components.

	Recognizing social norms			Resistance to framing			Applying decision rules			Consistency in risk perception		
	Total	Women	Men	Total	Women	Men	Total	Women	Men	Total	Women	Men
Age	0.03	0.08	-0.02	0.01	-0.02	0.04	-0.08**	-0.05	-0.14*	-0.04	-0.02	-0.07
Honesty-Humility	0.22**	0.18**	0.24**	0.02	-0.05	0.16**	0.14**	0.15**	0.17**	0.17**	0.19**	0.13*
Emotionality	0.09	0.14**	-0.01	-0.08	-0.04	-0.11*	0.01	0.12**	-0.10	0.07	0.13**	-0.05
Extraversion	0.03	0.06	-0.04	-0.12**	-0.11*	-0.11*	-0.02	0.04	-0.13*	0.02	0.03	-0.01
Agreeableness	0.01	0.03	-0.01	0.01	0.01	-0.02	-0.10**	-0.10*	-0.14*	0.00	-0.03	0.05
Conscientiousness	0.23**	0.20**	0.26**	-0.01	-0.04	0.07	0.28**	0.32**	0.26**	0.21**	0.20**	0.20**
Openness to experience	0.16**	0.17**	0.15*	-0.02	-0.05	0.05	0.16**	0.20**	0.14*	0.12**	0.11*	0.11*

*^∗^p < 0.05, ^∗∗^p < 0.01.*

### Measurement Invariance Testing Across Gender

Prior to examining the degree to which the HEXACO dimensions uniquely accounted for variance in overall DMC scores, we first sought to determine the degree of measurement invariance by means of a multiple group analysis (men/women) of a one-factor CFA with the four DMC variables as indicators of the latent variable. These analyses were conducted using the MPlus 7.4 (Muthén and Muthén, Muthén and Muthén) software package. Determining measurement invariance (i.e., the degree to which the factor loadings and intercepts of the latent variable are equivalent across groups) is a vital first step before comparing groups; if non-invariant, direct group comparisons may be inaccurate. Ideally, a construct should demonstrate scalar invariance (i.e., the factor loadings and intercepts should largely be equivalent; Vandenberg and Lance, 2000). However, strict measurement invariance rarely holds in practice. Best practices suggest that a latent construct should achieve at least partial invariance (i.e., an intercept of an indicator may be allowed to be freely estimated for each group, provided that a substantial drop in model fit is not observed; Byrne et al., 1989).

These results are shown in Table 3. Our first test, a comparison between configural (i.e., test of whether the structural CFA model is equivalent for men and women) and metric (i.e., factor loadings were the same across groups, but intercepts allowed to vary) invariance, yielded a non-significant chi-square difference test, Δχ^2^(6) = 12.09, *p* > 0.01. Further, the metric invariance model reasonably fit the data, suggesting that DMC across gender was metric invariant. However, when comparing the model fit based on a chi-square difference test between the metric and scalar (i.e., both factor loadings and intercepts are constrained to be equal across groups) invariance models, this value was significant Δχ^2^(3) = 21.15, *p* < 0.01. Upon inspection of the modification indices, we conducted a third model which relaxed the constraints on the Applying Decision Rules indicator, allowing the intercepts to vary across groups. This model yielded acceptable fit indices; the χ^2^ difference test between the partial and metric invariance model was not significant at *p* < 0.01, Δχ^2^(2) = 7.39. Our results suggest that partial measurement invariance across gender was established for the DMC construct.

**Table 3:** Fit statistics for multigroup analysis.

Model	*X*^2^	Df	*X*^2^*/*df	BIC	CFI	TLI	SRMR	RMSEA	RMSEA CI 90%
Configural invariance	1.62	2	0.81	8915.38	1.00	1.00	0.010	0.000	[0.000; 0.066]
Metric invariance	13.71	8	1.71	8942.68	0.972	0.958	0.048	0.042	[0.000; 0.079]
Scalar invariance	34.84	11	3.16	8943.76	0.884	0.873	0.060	0.073	[0.047; 0.102]
Partial invariance	21.12	10	2.11	8938.73	0.946	0.935	0.054	0.053	[0.020; 0.084]

*BIC, Bayesian Information Criterion; CFI, Confirmatory Fit Index (≥0.90); TLI, Tucker–Lewis Index (≥0.90); SRMR, Standardized Root Mean Square Residual (≤0.08); RMSEA, Root Mean Square Error of Approximation (≤0.08).*

### Testing the Unique Predictive Power of HEXACO Traits on DMC

**FIGURE 1:**
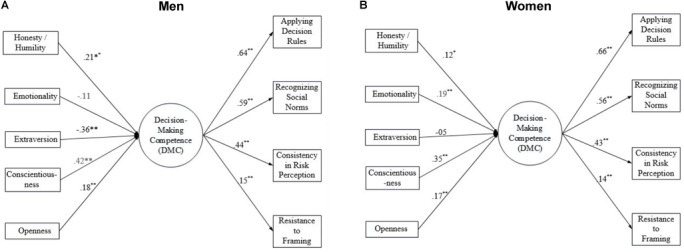
Multiple group analysis- standardized path estimates for men **(A)** and women **(B)**. CFI, Confirmatory Fit Index; TLI, Tucker–Lewis Index; RMSEA, Root Mean Square Error of Approximation; SRMR, Standardized Root Mean Square Residual). CFI = 0.924; TLI = 0.901; RMSEA = 0.042 (90% CI = 0.024–0.058); SRMR = 0.039.

Continuing with a multiple group analysis, we proceeded to add the HEXACO dimensions into the model as covariates in order to test the degree to which these traits accounted for individual differences in DMC. Although testing gender-specific paths between HEXACO and DMC should be considered exploratory, this analysis can shine light on potential interaction effects, while also controlling for gender differences in the intercept for Applying Decision Rules. Parameters were freely estimated using the maximum likelihood method. Our starting model included paths regressing the DMC latent variable on the six HEXACO dimensions and gender. In order to improve model fit, we removed Agreeableness from the model due to non-significant path coefficients to the DMC latent variable and re-ran the model. We examined modification indices to determine whether any direct effects from the personality dimensions to the DMC indicators would improve model fit (Muthén, 1989). Based on the modification indices, we added a direct path to Resistance to Framing scores from Extraversion and re-ran the model. Our final model reasonably fit the data (see Figure 1). Combined, the covariates accounted for 36.6 and 26.7% of the variance in DMC performance for men and women, respectively.

Consistent across gender, we found that three HEXACO dimensions accounted for heterogeneity in DMC scores. Conscientiousness was the strongest and most positive predictor of overall DMC performance. Additionally, Honesty/Humility and Openness were also both significantly, and positively, related to DMC scores. These findings align with our hypotheses. However, we found a divergent pattern for men and women for Extraversion and Emotionality, respectively. As shown in Figure 1A, introverted men scored higher on DMC than extroverted men. However, this association was not observed in women (see Figure 1B). A Wald test of parameter constraints confirmed that these effects were significantly different, χ^2^ = 11.67, *p* < 0.001. Additionally, for women, we found that greater emotionality was associated with greater DMC performance (Figure 1B). In contrast, there was a negative, albeit non-significant, path observed for men, Wald χ^2^ = 10.16, *p* = 0.001. The path coefficients for the other HEXACO dimensions did not yield a significant difference across gender.

The results from the multiple group analyses for Extraversion were stronger in magnitude than those observed with respect to the zero-order correlations with the DMC components, suggesting a potential suppressor effect. Considering that Conscientiousness and Openness both were positively correlated with Extraversion, and also positively associated with DMC, we suspected that the suppression effect occurred as a result of including either one, or both, of these variables. To confirm this, as a follow-up, we conducted a series of multiple regression analyses, first regressing DMC scores (calculated as a regression factor score of a one-factor exploratory factor analysis solution) on the six HEXACO dimensions. Then, we progressively removed the HEXACO dimension with the smallest non-significant standardized coefficient and re-ran the models to determine which variables need to be included for the suppression effect to remain. Only after removing both Conscientiousness and Openness (and leaving Extraversion as the sole predictor), we found that the standardized coefficient for Extraversion did not significantly predict DMC scores (β = -0.10, *p* = 0.09), compared to the coefficient when the other two variables remained in the model (β = -0.25, *p* < 0.001), or either one was removed (β = -0.19, *p* < 0.01, and -0.20, *p* < 0.001, for Conscientiousness and Openness, respectively). These findings suggest that the non-overlapping variance in Extraversion not attributable to Openness or Conscientiousness was associated with lower DMC scores, whereas the overlapping shared variance is associated with traits that promote better performance.

## Discussion

Accumulating research has demonstrated that individual differences in rational responding are associated with outcomes that are believed to be indicative of poor decision quality (Parker and Fischhoff, 2005; Bruine de Bruin et al., 2007; Stanovich et al., 2017; Toplak et al., 2017). However, less research has investigated the degree to which broad personality traits may be associated with these objective measures, despite documented associations between personality and similar life outcomes. In this study, we found that, irrespective of gender, three primary personality dimensions were associated with rational decision-making tendencies: Conscientiousness, Honesty/Humility, and Openness. Our analyses also revealed several gender-specific patterns of association; namely, an inverse relationship between Extraversion and DMC for men, and a positive association between Emotionality and DMC for women. These results were present even after controlling for small, but significant gender differences in performance on DMC component scales. Taken together, such findings extend our knowledge about both the construct validity of the DMC, as well as the functions of broad-based personality traits.

### Personality and DMC

As predicted, we found that Conscientiousness was the strongest positive predictor of DMC performance. Prior research has shown that Conscientiousness is a protective factor for desisting from health-risking behaviors, as well as being a predictor of academic and job performance, domains in which decision quality is likely to impact (Robbins et al., 2004; Terracciano and Costa, 2004). One implication of the current findings is that conscientious individuals may perform better in workplace settings (Schmidt and Hunter, 1998) because they are more self-regulated with respect to goal-oriented behavior. They are more likely to carefully evaluate options, maintain focus on set goals, filter irrelevant information, and effectively integrate multiple sources of information. Likewise, these skills may lead to an increased likelihood to respond rationally to decisions, which, in turn, are more likely to translate into advantageous long-term behavioral outcomes (e.g., Roberts et al., 2007).

Our results also support past research implicating Openness in rational decision-making. A core functional characteristic of Openness is the tendency to seek out and consider new knowledge when making choices. These tendencies are likely to contribute to competent decision-making by identifying a larger set of relevant information by which subsequent choices can be made. Beyond taking a broader perspective toward decisions, individuals scoring higher in Openness may feel intrinsically rewarded for engaging in complex thought, suggesting a motivational factor for responding in an accurate or consistent manner (Denissen and Penke, 2008). More broadly, our findings are consistent with research that has examined individual differences in Actively-Open-minded Thinking (AOT), a construct that shares overlapping variance with both Conscientiousness and Openness. This broad construct has been associated with higher performance on a variety of measures related to rational responding (Stanovich and West, 1997, 2007; Toplak et al., 2017). For example, Kokis et al. (2002) found that AOT scores predicted inductive and deductive reasoning performance of children as young as 10 years old. Our findings suggest that AOT could be decomposed in order to better understand the differential functions of its underlying components with respect to decision-making tendencies.

Additionally, these results add to the burgeoning evidence for the utility of assessing the Honesty/Humility dimension in addition to the traditional “Big 5” traits. By definition, a core component of low Honesty/Humility is the tendency for avoiding acting upon egoistic, self-interested impulses to the detriment of others – and often for short term gain. Consistent with this reasoning, we believe that Honesty/Humility is associated with low self-regulatory tendencies which may undermine advantageous decision-making (Lauriola and Weller, 2018). This myopic pattern of behavior has been observed in those reporting lower Honesty/Humility, and may involve the discounting of information (e.g., potential negative consequences, the feelings of others, etc.) in favor of perceived personal gain, evidenced by counterproductive work and academic behaviors. It can also be observed in an elevated tendency to report health and ethical risk taking. Weller and Tikir (2011) found that the relationship between Honesty/Humility and risk taking in these domains were mediated by reductions in risk perceptions and increases in perceived expected benefits associated with those activities. We believe that this finding suggests a less normative evaluation of potential dangers and rewards, which could also be viewed as an indicator of lower DMC.

We acknowledge that, on the surface, our results might appear to run counter to research highlighting that individuals who score high in Honesty/Humility show greater cooperation on certain behavioral economics tasks, such as the Ultimatum Game (Hilbig and Zettler, 2009; Zhao and Smillie, 2015). Hilbig and Zettler (2009) interpreted this finding as being indicative of *less* rational behavior. Although this prior finding was interpreted as evidence of irrationality, it must be noted that claims of rationality on this task may only be invoked if the actor is actually aiming to maximize the subjective utility function of the *payoff amount* of the game. From an evolutionary game theory perspective, Nowak et al. (2000) argue that individuals who reject more unfair offers may be maximizing reputation, especially if they expect future interactions. It is tenable to reason that those reporting greater Honesty/Humility are individuals who may strongly value fairness and sincerity; hence, they may be more likely to maximize reputation rather than payouts, making their response rational within utility maximization context. Nonetheless, we find this divergence interesting and hope that future research may further disentangle these findings.

### Gender Differences in DMC

Our results also help to clarify the nature of gender differences for DMC, in which prior research has been mixed. Whereas some studies have shown that men perform better on metrics of rational responding such as expected value sensitivity and probabilistic reasoning, other research using different decision-making paradigms have shown no differences (Stanovich et al., 2017). The current results suggest that observed gender differences may be more confined to a range of particular subcomponents, rather than global differences. The measurement invariance analyses illustrated this point. Specifically, our results suggested that a one-factor solution reasonably fits the data for both men and women, though the results did not reveal full scalar invariance. Specifically, men and women responded differently to the Applying Decision Rules scale, suggesting that overall DMC scores for women may be associated with poorer performance within a particular subdomain, rather than global differences across all forms of rational responding. However, partial invariance is viewed as being adequate for comparing latent variable scores across groups, and suggests that a common regression line, regressing a criterion on DMC, can be used. However, this point should be taken into consideration in future studies when comparing other groups, as it may impact the interpretability of DMC and related constructs as a predictive measure.

Notably, we observed two gender-specific associations between HEXACO traits and DMC performance. First, we found that higher Emotionality was associated with better decision-making for women only. This finding was surprising, considering past research that has demonstrated that anxiety and fear, other components of Emotionality, may impair advantageous decision making and heighten risk perceptions (Lerner and Keltner, 2001; Miu et al., 2008; de Visser et al., 2010; Weller and Tikir, 2011). However, it is also important to note that the HEXACO Emotionality scale contains more positive aspects than those typically included in Neuroticism/Negative Emotionality scales. For instance, it includes items related to Sentimentality, or the tendency to create emotional bonds with others. We tentatively speculate that this facet may promote skills associated with advantageous decision-making. For example, developing and maintaining interpersonal emotional bonds requires listening to others and considering alternative viewpoints, two skills that are important for making long-term advantageous decisions. However, this explanation does not fully address why greater Emotionality was associated with better performance for the other DMC scales. Considering that we did not have an a priori hypothesis for this effect, nor could we find any supporting theoretical explanations, future research designed to more extensively test the associations between facet-level Emotionality (which we could not do reliably with the HEXACO-60) and a broader range of decision-making skills is needed in order to attain greater clarity. At the very least, though, our findings point to the promise of a more nuanced view of Emotionality, highlighting that more positive aspects of emotionality may be associated with better overall decision-making skills.

Another unexpected finding was that higher Extraversion was associated with lower DMC scores for men, but not women. According to the cybernetic Big Five model, Extraversion may represent approach motivations and is posited to be particularly sensitive to potential rewards (DeYoung, 2015). The current results are intriguing because, with the exception of some risky-choice framing items, reward sensitivity would not be expected to be a primary driver of choice behavior across these tasks in the same manner as in a risk-taking task, for example. Notably, though, Resistance to Framing was the only DMC scale that demonstrated significant zero-order correlations with Extraversion across gender.

Our follow-up analyses revealed that, for men, the effects of Extraversion became stronger when simultaneously accounting for Conscientiousness and Openness. This finding suggests that the overlapping variance shared by these three dimensions has a positive effect on DMC, whereas Extraversion’s unique component(s) has a mitigating effect. One potential source of this non-overlapping variance may be sensation-seeking tendencies, which are related to increased risk taking over a wide context of behaviors (de Vries et al., 2009; Lauriola and Weller, 2018). Though risk-taking, from a behavioral economic perspective, does not necessarily imply irrational decision processes, consistently engaging in behaviors that have the potential to bear significant adverse consequences to health, financial, and social well-being can be considered maladaptive and indicative of poor decision quality (see Dewberry et al., 2013). Notably, gender-specific associations between risk behaviors and sensation-seeking previously have been reported. For instance, Zuckerman and Kuhlman (2000) reported higher correlations between sensation-seeking and certain risk behaviors (i.e., gambling and drug use) for men in comparison to women. Unfortunately, like the gender-specific pattern observed with Emotionality, we cannot adequately test this hypothesis with the 60-item HEXACO; thus, we underscore that these assertions are speculative, and future research must be conducted to better understand these effects.

### Avenues for Future Research

We acknowledge several directions for future research. First, despite a large sample size, we obtained a low response rate for the study sample. As a consequence, we must temper our conclusions about the degree to which we can generalize these findings to the broader population. One potential problem of low response rate is that individuals higher in DMC self-selected to take part in this study. Nevertheless, both all the HEXACO dimensions and a composite DMC score created by the factor score method approached a normal distribution, which would speak against range restriction as a potential problem. Though we cannot rule out mean-level differences in DMC scores between responders and non-responders, we have no reason *a priori* to believe that the observed correlations between personality and DMC would differ in a representative sample.

Second, because of time constraints, we assessed shorter versions of both the A-DMC measures, as well as the HEXACO. As a result, our study is silent to the associations between personality and DMC components such as Over/Underconfidence and Resistance to Sunk Costs. Notably, the overconfidence scale did not correlate with other DMC scales in the only paper reporting these associations with the full Italian-language version of the DMC battery, suggesting potential cross-cultural variation (such as items related to interactions in relationships; Del Missier et al., 2012). Thus, we hesitated to use this version of the test without invariance testing of its own, which speak beyond our data. As researchers from other countries begin to adopt and use the DMC measure, testing cross-cultural non-invariance is a vital goal so that researchers can compare results across different samples with confidence that these scales measure the same construct(s). However, even in its full length, the A-DMC battery only includes six tasks for assessing decision-making tendencies. For instance, the A-DMC measure does not include assessments of intertemporal choice, “classic” heuristics and biases (e.g., representativeness and availability heuristics; Kahneman and Tversky, 1972; Ceschi et al., 2018), or the ability to make expected-value sensitive choices (Parker and Weller, 2015; Stanovich et al., 2017). Integrating other decision-making skills into a DMC framework and further explicating its construct validity should be a continual psychometric goal of future research (for a laudable effort in this direction, see Stanovich et al., 2017).

As a final caveat, some of the reported correlations were small in absolute magnitude. We caution readers to not simply categorize small effects as trivial (Cortina and Landis, 2009). This caveat is especially important to acknowledge because associations between broad trait dimensions and single instances of behavior are likely to be lower than if one aggregated assessments of DMC over time. Additionally, DMC performance is multifarious in nature, and thus, it is unlikely that any one broad trait dimension will account for a proportion of the variance that would be considered a “strong” effect size (Cohen, 1994). As we have previously noted, future studies may benefit from a more thorough investigation of traits at the facet-level. We acknowledge a tradeoff between bandwidth and fidelity, in which broader traits may be able to predict a larger range of outcomes, but more narrow traits (e.g., facets) may predict more specific behaviors in a superior manner (Cronbach and Gleser, 1957). This tradeoff also may be important in functional personality frameworks because lower-level traits may represent more specific functions related to the broader functions of higher-order traits (DeYoung, 2015).

## Conclusion

Because of the descriptive nature of traits like the Big 5, theoretical discussion about their functional utility often is less highlighted. That is, individual differences research is often clear about *who* engages in a behavior rather than *why* (i.e., mechanisms that may underlie behaviors). In summary, the current study provides insights into how personality may be associated with decision-processes. More broadly, the current results represent a step in linking broader self-regulatory functions, represented by trait dimensions, with more fine-grained decision-making mechanisms. We hope that this study will help to guide future research that aims to understand the underlying mechanisms in what ways traits may impact behavior. By bridging descriptive personality research associated with social-cognitive mechanisms, researchers may begin to more fully appreciate the cognitive and behavioral tendencies of those who possess different levels of broader trait dimensions.

## Ethics Statement

This study was carried out in accordance with the guidelines followed by the Ethical Review Committee at the University of Verona, with written (via the internet) informed consent from all subjects. All subjects gave informed consent electronically in accordance with the Declaration of Helsinki. The protocol was approved by the Ethical Review Committee at the University of Verona.

## Author Contributions

The authors discussed the contents of this article together. JW and AC developed the research hypotheses, devised the methodological content, and analyzed the data. LH, RS, and AC conferred with JW and AC about theoretical and empirical aspects of the study and provided a significant contribution to the interpretation and discussion of the research findings. The final version of the article was written by JW, AC, LH, RS, and AC.

## Conflict of Interest Statement

The authors declare that the research was conducted in the absence of any commercial or financial relationships that could be construed as a potential conflict of interest.

## Footnotes

1 Expected-value sensitivity indicates that an individual chooses an option with a more favorable expected value (expressed as Σ EV = probability ^∗^outcome magnitude of each possible option), given at least two option. Making EV-sensitive choices can be considered an index of advantageous decision-making because, mathematically, choosing an option with a superior EV consistently should lead to more favorable outcomes over the long-run.

2 The scoring procedure used in this study is a simpler version of scoring for Consistency in Risk Perception scale than the original method, which also includes consistency across subsets/supersets and complementary events. We chose this to maintain consistency with prior research using the same dataset (Weller et al., 2015b). For the sake of due diligence, we compared the Consistency of Risk Perception scale, as we calculated it, with the Bruine de Bruin et al. (2007) method. Our analyses robustly indicated that the results with the “expanded” scored scale strongly resembled those currently in the paper. Specifically, we found (a) no significant gender differences, (b) *r* = 0.88 between the two measures; (c) *r* = 0.97 the composite factor scores for one-factor solutions, and (d) correlation values between the expanded composite and the one currently in the scale were highly similar, only changing by at most 0.01. We note that this simplified manner by which this scale has been calculated has been utilized before using other versions of the DMC battery, with similar results (Parker and Fischhoff, 2005; Weller et al., 2012).

3 http://hexaco.org
