# Supplementary material for: Accounting for Individual Differences in Decision-Making Competence: Personality and Gender Differences
Source: Front Psychol. 2018 Nov 23;9:2258. doi: 10.3389/fpsyg.2018.02258 (PMC6276324; doi:10.3389/fpsyg.2018.02258)
Supplement: Supplementary file 1 [file Table_1.docx]

**Supplementary Information**

S1. A-DMC intercorrelation matrix.

|  | 1 | 2 | 3 | 4 |
| --- | --- | --- | --- | --- |
| 1. Recognizing Social Norms | -- |  |  |  |
| 1. Applying Decision Rules | **.36** | -- |  |  |
| 1. Consistency in Risk Perception | **.27** | **.25** | -- |  |
| 4. Resistance to Framing | .09* | **.13** | .06 | -- |

*Note. N* = 804. Correlations in bold are significant at *p*<.01.**p* <.05. Previously

reported in Weller et al., 2015.

S2. Intercorrelations between HEXACO dimensions

|  | 1 | 2 | 3 | 4 | 5 | 6 |
| --- | --- | --- | --- | --- | --- | --- |
| 1. Honesty/Humility | -- |  |  |  |  |  |
| 2. Emotionality | **.36** | -- |  |  |  |  |
| 3. Extraversion | **.27** | **.25** | -- |  |  |  |
| 4. Agreeableness | .09* | **.13** | .06 | -- |  |  |
| 5. Conscientiousness |  |  |  |  | -- |  |
| 6. Openness |  |  |  |  |  | -- |

Note. *N* = 804. Correlations in bold are significant at *p*<.01.**p* <.05.
